# Complete variable domain sequences of monoclonal antibody light chains identified from untargeted RNA sequencing data

**DOI:** 10.3389/fimmu.2023.1167235

**Published:** 2023-04-18

**Authors:** Allison Nau, Yun Shen, Vaishali Sanchorawala, Tatiana Prokaeva, Gareth J. Morgan

**Affiliations:** ^1^ Amyloidosis Center, Boston University Chobanian & Avedisian School of Medicine, Boston, MA, United States; ^2^ Research Computing Services, Boston University, Boston, MA, United States; ^3^ Section of Hematology and Medical Oncology, Department of Medicine, Boston University Chobanian & Avedisian School of Medicine, Boston, MA, United States; ^4^ Department of Pathology and Laboratory Medicine, Boston University Chobanian & Avedisian School of Medicine, Boston, MA, United States

**Keywords:** antibody sequence, AL amyloidosis, multiple myeloma, plasma cell dyscrasia, antibody light chain, monoclonal gammopathy, MiXCR, antibody repertoire sequencing

## Abstract

**Introduction:**

Monoclonal antibody light chain proteins secreted by clonal plasma cells cause tissue damage due to amyloid deposition and other mechanisms. The unique protein sequence associated with each case contributes to the diversity of clinical features observed in patients. Extensive work has characterized many light chains associated with multiple myeloma, light chain amyloidosis and other disorders, which we have collected in the publicly accessible database, AL-Base. However, light chain sequence diversity makes it difficult to determine the contribution of specific amino acid changes to pathology. Sequences of light chains associated with multiple myeloma provide a useful comparison to study mechanisms of light chain aggregation, but relatively few monoclonal sequences have been determined. Therefore, we sought to identify complete light chain sequences from existing high throughput sequencing data.

**Methods:**

We developed a computational approach using the MiXCR suite of tools to extract complete rearranged *IGV_L_-IGJ_L_* sequences from untargeted RNA sequencing data. This method was applied to whole-transcriptome RNA sequencing data from 766 newly diagnosed patients in the Multiple Myeloma Research Foundation CoMMpass study.

**Results:**

Monoclonal *IGV_L_-IGJ_L_* sequences were defined as those where >50% of assigned *IGK* or *IGL* reads from each sample mapped to a unique sequence. Clonal light chain sequences were identified in 705/766 samples from the CoMMpass study. Of these, 685 sequences covered the complete *IGV_L_-IGJ_L_* region. The identity of the assigned sequences is consistent with their associated clinical data and with partial sequences previously determined from the same cohort of samples. Sequences have been deposited in AL-Base.

**Discussion:**

Our method allows routine identification of clonal antibody sequences from RNA sequencing data collected for gene expression studies. The sequences identified represent, to our knowledge, the largest collection of multiple myeloma-associated light chains reported to date. This work substantially increases the number of monoclonal light chains known to be associated with non-amyloid plasma cell disorders and will facilitate studies of light chain pathology.

## 1 Introduction

Aberrant proliferation of clonal, antibody-secreting plasma cells in the bone marrow causes a spectrum of disorders known as plasma cell dyscrasias (PCDs), which include multiple myeloma (MM), amyloid light chain (AL) amyloidosis and other “monoclonal gammopathies of clinical significance” (1–3). Monoclonal antibody light chains (LCs) secreted from these aberrant plasma cells without a heavy chain partner are known as free light chains (FLCs). These FLCs can form diverse aggregate structures in multiple tissues, leading to progressive tissue damage, organ failure and death if untreated (1, 4–6). Three major forms of aggregate are renal tubular casts, where FLCs form co-aggregates with uromodulin (Tamm Horsfall protein) (7); unstructured deposits, observed in light chain deposition disease and related disorders (6); and amyloid fibrils, which are highly ordered arrays of LC-derived peptides in a non-native conformation (8). However, the majority of individuals with a detectable monoclonal antibody or FLC in circulation do not have evidence of amyloid formation or other LC pathologies when the PCD is identified (9), consistent with the hypothesis that only a subset of FLCs can form pathological aggregates *in vivo*. FLC aggregation is therefore hypothesized to be a function of both the unique sequence of the monoclonal LC and its level in circulation. The diversity of the antibody repertoire—there are an estimated 10^6^-10^7^ LC sequences in a healthy human, with some overlap between individuals—makes it difficult to identify which features of a clonal LC protein contribute to pathology (10, 11). Mechanistic understanding of how LC sequence features drive FLC aggregation could lead to new therapies and potentially allow these toxic LCs to be identified before the onset of symptoms. To this end, the sequences of many unique monoclonal LCs from different PCDs have been determined. Here, we present an approach to identify monoclonal LC sequences from new or existing RNA sequencing data.

Each PCD clone expresses a unique LC protein (12, 13). Functional antibody LCs are encoded by immunoglobulin (IG) genes, comprising a variable (*IGV_L_)*, joining (*IGJ_L_*) and constant (*IGC_L_*) fragment. These fragments are assembled by VJ recombination from germline gene precursors during B cell development, followed by somatic hypermutation and selection for antigen affinity (14). There are two types of LCs, kappa (κ) and lambda (λ), each encoded by an independent locus in humans: *IGK* on chromosome 2 and *IGL* on chromosome 22. In this report, the rearranged genes are referred to as *IGV_L_-IGJ_L_* sequences, which includes both κ and λ LCs. Where the type of rearrangement is known we refer to *IGKV-IGKJ* and *IGLV-IGLJ* sequences.

**Figure 1:**
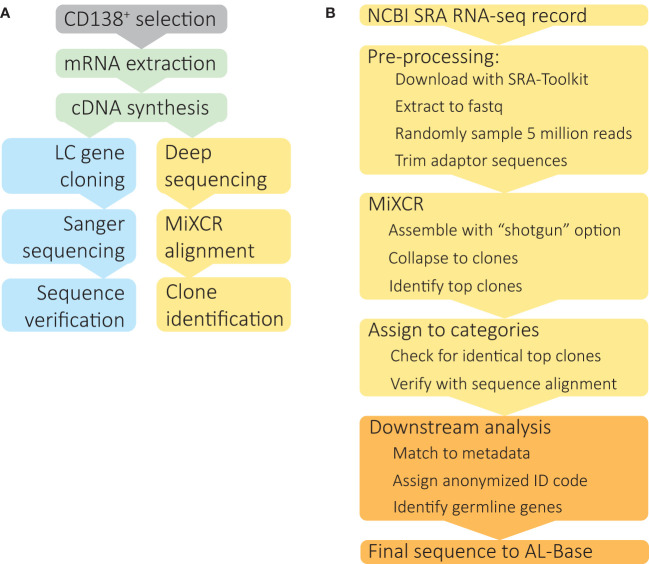
Identification of clonal *IGV_L_-IGJ_L_* sequences from untargeted RNAseq data. **(A)** Schematic depiction of *IGV_L_-IGJ_L_* sequence determination methods. Following optional enrichment of CD138+ plasma cells, total mRNA is extracted and cDNA synthesized by reverse transcription. Standard *IGV_L_-IGJ_L_* cloning methods (blue boxes) use specific primers to amplify coding regions, followed by Sanger sequencing and validation by PCR, or, more recently, by high throughput sequencing approaches. The method described here (yellow boxes) takes deep sequencing datasets acquired for gene expression studies and uses the MiXCR suite of tools to identify clonal *IGV_L_-IGJ_L_* sequences. **(B)** Computational analysis of RNAseq data to identify complete *IGV_L_-IGJ_L_* sequences, using software tools described in the Methods. The steps shown in yellow boxes are automatic and require only the SRA accession as an input; the output from each step is passed to the next program. Downstream analysis and deposition in AL-Base, shown in orange, requires manual oversight.

A monoclonal LC’s protein sequence defines its structure and biophysical properties and hence its propensity to aggregate and cause disease (15). Monoclonal immunoglobulin sequences can be cloned and sequenced from bone marrow samples (**Figure 1A**), but the established procedure is slow and labor-intensive (16, 17). Cloning of individual genes therefore represents a significant barrier to studying LCs at scale, although emerging methods are increasing the rate of sequence discovery using targeted amplification and high throughput sequencing technologies (18, 19). Although MM is the most common symptomatic PCD, relatively few MM-associated *IGV_L_-IGJ_L_* sequences have been determined. Such sequences could inform efforts to understand LC-mediated pathology in MM and also serve as controls for studies of aggregation propensity.

Sequences of disease-associated and other LCs are collected in the Boston University AL-Base resource, https://albase.bumc.bu.edu/aldb (12). Of 800 monoclonal PCD-associated LC sequences deposited in AL-Base prior to this study, 180 were associated with MM, compared to 527 associated with AL amyloidosis. We therefore sought to identify additional LC sequences associated with MM and develop a method by which new *IGV_L_-IGJ_L_* sequences from diverse sources could be identified.

High throughput sequencing techniques have transformed the study of antibody repertoires in recent years (20, 21). Targeted amplicon sequencing of antibody cDNA from B cells in blood, bone marrow or other tissues can yield millions of sequences per sample (10, 11, 21). Although individual sequencing reads are usually shorter than antibody transcripts, multiple reads can readily be assembled into contiguous sequences. Furthermore, antibody sequences can be identified from untargeted RNA sequencing (RNAseq) experiments, allowing the diversity of B cells within tissue samples to be estimated from a standard transcriptomic experiment (22). These analyses often focus on complementarity determining region 3 (CDR3), the most diverse stretch of antibody sequences, to enumerate and track related groups of sequences, known as clonotypes. For example, Rustad et al. showed that *IGV_L_-IGJ_L_* CDR3 region sequences could be used to identify clonal plasma cells in MM, and that these sequences are retained over the course of disease (23). Langerhorst et al. used a similar approach to identify peptides that could be tracked by mass spectrometry (24). However, neither study reported complete *IGV_L_-IGJ_L_* sequences, which would be needed to study the physicochemical properties of LCs.

We reasoned that clonal plasma cell samples isolated from bone marrow might yield sufficient RNAseq reads for identification of complete consensus *IGV_L_-IGJ_L_* sequences, which are needed for functional studies. This approach could bypass the time and effort required to clone genes individually (**Figure 1A**) and allow identification of clonal *IGV_L_-IGJ_L_* sequences from existing RNAseq-based gene expression studies. The MiXCR software platform can identify immune receptor sequences (rearranged B and T cell receptor genes) from untargeted RNAseq data (22), and assemble long contiguous nucleotide sequences from targeted repertoire sequencing (25), making it a suitable choice for this project.

Here, we establish a computational method to identify clonal, rearranged *IGV_L_-IGJ_L_* gene sequences from RNAseq data using the MiXCR software package (22, 25, 26). We applied this procedure to data from a large cohort of patients with newly diagnosed MM, the Clinical Outcomes in Multiple Myeloma to Personal Assessment of Genetic Profiles study (CoMMpass) run by the Multiple Myeloma Research Foundation (MMRF) (27). From 766 individual samples, 705 clonal *IGV_L_-IGJ_L_* sequences were identified, of which 685 covered the complete *IGV_L_-IGJ_L_* region. Our approach allows complete *IGV_L_-IGJ_L_* sequences to be identified from existing RNAseq datasets and may allow routine determination of monoclonal sequences as part of plasma cell gene expression experiments.

## 2 Materials and Methods

### 2.1 Datasets and patients

RNAseq data were obtained from CD138-enriched bone marrow samples that were derived from 766 MM cases from the MMRF CoMMpass trial (release IA15) at baseline evaluation. Each clinical case corresponds to a single RNAseq data file for the initial sample taken at diagnosis. RNAseq data were accessed *via* the NCBI database of Genotypes and Phenotypes (dbGaP) website *via* the Authorized Access system (accession phs000748.v7.p4). Details of the RNAseq experiments have previously been published (23). Briefly, polyadenylated RNA from CD138-selected bone marrow mononuclear cells was sequenced using 100 nt paired-end reads to a target depth of 100 million reads on the Illumina HiSeq platform. Clinical data were obtained from the MMRF Research gateway (http://research.themmrf.org) after registration and approval for data use. All patient data were deidentified and assigned a new random code to allow sequence deposition into the AL-Base repository (https://albase.bumc.bu.edu/aldb/).

### 2.2 Hardware and software

Large-scale computational analyses were run on the Boston University Shared Computing Cluster, comprising multiple nodes with Intel Xeon processors running Linux. A maximum of four processor cores and 64 Gb of RAM was requested for each operation. Jobs were scheduled using the Sun Grid Engine (Oracle, USA) queuing system. RNAseq data were downloaded using sratoolkit (v2.11.1) (28). Sequence quality was checked using FastQC (v0.11.7) (29) and MultiQC (v1.10.1, Python v3.7.9) (30). Sequence reads were downsampled using seqtk (v1.3) (31), and adapters were trimmed using trimmomatic (v0.39) (32). Antibody sequences were identified and assembled with MiXCR (v3.0.13) (22, 25, 26). Since the time of analysis, a major upgrade to MiXCR (v4.1.0) has been released, focused on single cell and barcoded data, but the core functionality remains the same and v3.0.13 is available and free for non-profit use at https://github.com/milaboratory/mixcr/releases/. Processed data were analyzed and summary statistics calculated using R (v.4.0.5) (33) *via* the RStudio interface (34). The following packages were used: Biostrings (35), Tidyverse (36), msa (37), stringr (38), janitor (39), scales (39, 40), ggpubr (41), cowplot (42), naniar (43), rstatix (44), moments (45), epitools (43, 46), and gtools (47). Some data processing was performed in Python (v3.8.10 unless otherwise specified) (48) using the standard library and the packages pandas (v1.2.4) (49), NumPy (v1.19.5) (50) and Biopython (v1.78) (51). Pairwise alignments were created using NCBI BLAST+ (v2.12.0) (52) and highly related sequences were collapsed into a single sequence using CAP3 (53).

Scripts for data processing are available at Github: https://github.com/buamyloid/lightchain-from-rnaseq

### 2.3 Overview of RNAseq analysis

The steps in our analysis are illustrated in **Figure 1B**. For each individual case, RNAseq data were downloaded from the Sequence Read Archive (SRA) and converted to fastq format using sratoolkit. Sequence quality was assessed using FastQC and MultiQC, which could potentially be used to diagnose problems with later steps. A random sample of 5 million paired end reads was used for analysis. Adaptor sequences were removed using trimmomatic. Reads were aligned to the *IGH*, *IGK* and *IGL* loci and assembled into clonal sequences using MiXCR. From the MiXCR output, the major clonal *IGV_L_-IGJ_L_* sequence was identified. *IGH* reads are assembled in this process but not considered further in this study. The fraction of reads assigned to each clone, which are referred to in MiXCR as “counts”, and the length of the contiguous nucleotide sequence were determined. In some cases, two identical and overlapping clonotypes were identified, which could be collapsed to a single, longer sequence following manual verification of the sequence alignment. Based on the fraction of counts assigned to the major clone, each sample was assigned to a category. Samples with a clone that accounted for more than 50% of assigned counts and covered the complete *IGV_L_-IGJ_L_* region were accepted as the final clonal sequence.

### 2.4 Validation of MiXCR performance in identification of correct *IGV_L_-IGJ_L_* transcripts

To test the feasibility of this approach in identification of *IGV_L_-IGJ_L_* transcripts, the clonotypic sequence identified by MiXCR was compared to the sequence determined by standard cloning methods. For this purpose, untargeted RNAseq data from the U266 MM cell line (SRA reference GSM2334829) (54) and previously reported U266 *IGLV2-8-IGLJ2-IGLC2* sequence (55) were used.

### 2.5 MiXCR analysis

Data processing was optimized using three samples from the CoMMpass study. Individual samples typically yielded 10-100 million paired end reads in a single FASTQ file. Following adaptor trimming, 80 to 90 nucleotides remained on each of the paired reads. MiXCR analysis of large input files required long processing times, which may be impractical for some applications. Therefore, we randomly sampled a subset of reads from each file to use as the input to MiXCR. From each of three files, triplicate independent subsets of 100K, 200K, 500K, 1M, 2M, 5M, and 10M reads were randomly sampled using seqtk (31) and processed with MiXCR. By default, MiXCR applies a quality control filter to input reads. Accordingly, removal of low-quality nucleotides from the input did not affect the final output or processing speed.

MiXCR analysis was run using the “assemble shotgun” command, which is optimized for untargeted RNAseq data (22). Each input FASTQ file, corresponding to a single clinical case, was processed independently. Only “BCR” (B cell receptor) alignments were specified, so MiXCR reported clonal sequences derived from the *IGH*, *IGK* and *IGL* loci. We distinguish here between input “reads” from the sequence data and “counts”, the number reads assigned to each identified clonotype. All calculations of clonal fractions are based on counts, rather than input reads. MiXCR uses a clustering algorithm to collapse reads to contiguous clonotypes. The counts assigned to subclusters of sequences, referred to by MiXCR as “children” were included in the total counts assigned to each cluster. Otherwise, default options were used for assignment and assembly procedures. The output from MiXCR is a list of clonal sequences and the number of individual counts from the sample that contributed to each sequence. The *IGV_L_-IGJ_L_* sequence with the most assigned counts from each clinical case was defined as the clonal sequence. For some cases MiXCR returns a non-contiguous sequence from the most frequent clone. In the analyses below, the longest fragment identified by MiXCR was used.

### 2.6 Assignment of clones to categories

To evaluate MiXCR’s performance in defining a unique clone for each clinical case, the output from each processed sample was classified into one of four categories based on the fraction of total *IGK* and *IGL* counts assigned to the most frequent clone. In some cases, MiXCR identified two overlapping clones with identical sequences, which we attempted to collapse into a single clonal sequence. Only samples where a second clone from the same locus with ≥100 counts, accounting for >1% of the counts from the *IGL* or *IGK* clones, were considered for analysis. The identity of these clones was verified by pairwise alignment using NCBI BLAST+, *via* Biopython. The two clones were combined into one when they had an overlapping region of ≥200 nt, with 100% sequence identity over the overlap, and together accounted for ≥95% of total counts.

### 2.7 Germline gene assignment

Following identification of the major clones, the longest contiguous nucleotide sequence from each sample was aligned to the ImMunoGeneTics (IMGT) databases of human immunoglobulin genes using the NCBI IgBLAST tool (https://www.ncbi.nlm.nih.gov/igblast/) (56) and the IMGT HighV-QUEST tool (https://www.imgt.org/HighV-QUEST/home.action) (57). Identification of one *IGV_L_*, *IGJ_L_* and *IGC_L_* gene per sample was specified where necessary. Individual germline gene precursors follow the IMGT nomenclature with *IGKV*, *IGKJ*, *IGKC*, *IGLV*, *IGLJ*, and *IGLC* prefixes.

### 2.8 Validation of clonal *IGV_L_-IGJ_L_* sequences

To verify that each clinical case yielded a unique *IGV_L_-IGJ_L_* sequence, each clonal sequence was compared to all other sequences from the cohort at both the nucleotide and protein level, using text matching in R. Protein sequences identified by alignment to the IMGT databases were compared to those previously determined by Rustad et al. (23) and Langerhorst et al. (24) for the CoMMpass cohort data, using text matching in R.

### 2.9 Clinical data analysis

Baseline quantitative serum κ and λ FLC values as well as data on the presence of monoclonal immunoglobulin (M-protein) were obtained in the MMRF Research gateway clinical dataset (http://research.themmrf.org/). Patients enrolled in the CoMMpass study had FLC levels measured by immunoassay and M-protein was identified by immunofixation or serum protein electrophoresis at the initial visit, when bone marrow was sampled for RNAseq analysis. The κ/λ FLC ratios were calculated from the entries in the D_LAB_serum_kappa and D_LAB_serum_lambda fields and compared to the reference range of 0.26-1.65 (58) to determine whether each case was classified as κ- or λ-restricted at diagnosis. Of the 660 cases with available FLC data, 306 also had LC M-protein identified in the D_IM_IGL_SITE field. The identity of the most frequent clone identified by MiXCR was compared to the serum LC restriction.

For inclusion in AL-Base, AL amyloidosis was determined based on the entry in the SS_AMYLOIDOSIS field at baseline and follow-up clinical visits. A total of 25 cases with amyloidosis were reported in the CoMMpass IA15 data. Of these, 14 had baseline RNAseq data available for analysis.

## 3 Results

### 3.1 Clonal *IGV_L_-IGJ_L_* sequences from untargeted RNAseq data

**Figure 2:**
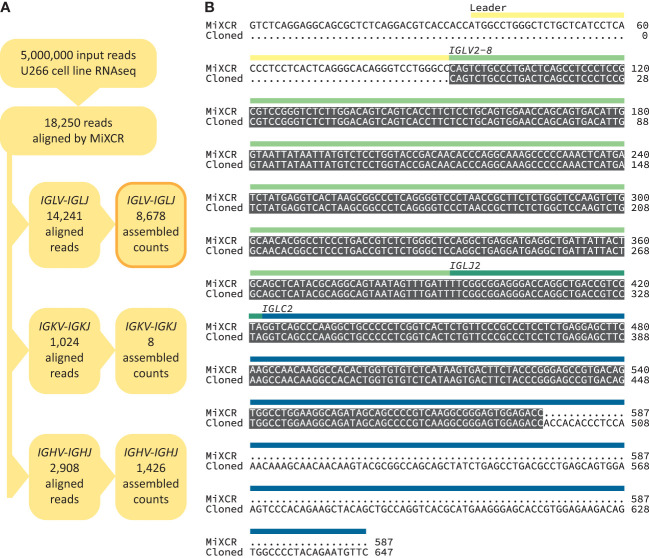
Accurate recovery of U266 *IGV_L_-IGJ_L_* sequence. **(A)** RNAseq reads aligned by MiXCR to immunoglobulin loci. **(B)** Alignment between U266 *IGLV2-8-IGLJ2-IGLC2* sequences derived from untargeted RNAseq data (54) using MiXCR and standard cloning methods (55). Identical regions are highlighted in grey. The regions of the monoclonal sequences are shown with yellow, green and blue bars.

We built a software analysis pipeline around MiXCR (25) to routinely identify *IGV_L_-IGJ_L_* sequences from RNAseq data derived from clonal plasma cells (**Figure 1B**). We first asked whether the sequences identified by MiXCR were identical to those determined by standard cloning methods, using public data from the U266 MM cell line (**Figure 2**). From an untargeted RNAseq experiment [SRA reference GSM2334829 (54)], MiXCR identified a single clonal *IGV_L_-IGJ_L_* sequence. The initial MiXCR step aligned 18,250 reads, 0.37% of the total input reads, to T cell receptor or B cell receptor genes. This was lower than the fraction of reads associated with immunoglobulins in plasma cells, because MiXCR requires at least one of each pair of reads to be aligned to the V(D)J junction region with high stringency. Of these aligned reads, 14,241 (78.0%), 1024 (5.6%), and 2908 (15.9%) were aligned to *IGL*, *IGK*, and *IGH* loci, respectively (**Figure 2A**). In the final MiXCR output, the counts of aligned reads that were successfully assembled were 8,678, 8, and 1,426 for the *IGL*, *IGK*, and *IGH* loci, respectively. MiXCR assembly of these reads yielded a clone derived from the *IGLV2-8*, *IGLJ2* and *IGLC2* precursor genes that accounted for 8614 of the successfully assembled reads, corresponding to 99.2% of MiXCR *IGL* or *IGK* count and 85.2% of the total MiXCR immunoglobulin count. This clonal sequence started upstream of the protein coding region and extended through the leader, *IGLV* and *IGLJ* regions, partially into the *IGLC* region. The MiXCR-derived sequence is identical to a *IGLV-IGLJ-IGLC* sequence previously cloned from the same cell line (55) over the region where the sequences overlap, which covers the entire *IGLV*-*IGLJ* region (**Figure 2B**). The low number of counts assigned and assembled by MiXCR to a unique *IGH* clone is consistent with the observation that U266 cells secrete only a FLC protein (55). We therefore conclude that analysis of bulk, untargeted RNAseq data with MiXCR can yield accurate *IGV_L_-IGJ_L_* transcripts.

### 3.2 Efficient clonal *IGV_L_-IGJ_L_* sequence identification from five million reads

**Figure 3:**
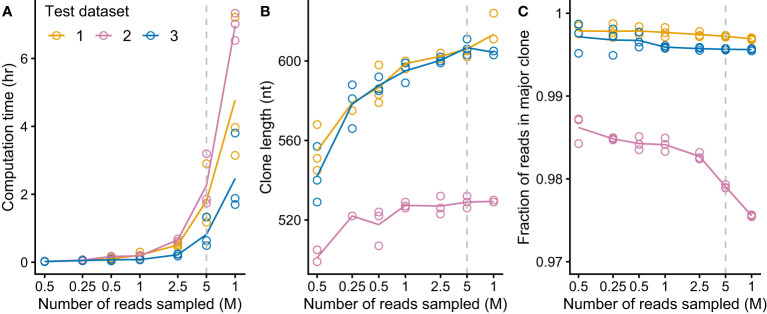
MiXCR identifies *IGV_L_*-*IGJ_L_* clones from an input of five million randomly sampled reads. For each of three CoMMpass cases, random samples of reads (three replicates each of seven sample sizes) were used as the input to MiXCR and the resulting clonal sequences were analyzed. Dashed lines show the results using 5M reads, which was chosen as the target for down-sampling. **(A)** Computation time for varying amounts of input reads, using four cores on an Intel Xeon processor. **(B)** Length of output top *IGV_L_-IGJ_L_* clone for varying amount of input reads. (For comparison, a typical *IGV_L_-IGJ_L_* sequence is approximately 330 nt.) For cases where the output from MiXCR comprised multiple non-contiguous segments for a single clone, only the longest segment was considered. **(C)** Fraction of counts assigned by MiXCR to the major *IGV_L_-IGJ_L_* clone.

We aimed to create a complete pipeline that would identify and evaluate clonal sequences without additional intervention, so that multiple samples could be processed efficiently. It was important to verify that clonal sequences could be identified from clinical samples as RNAseq data derived from these specimens is more complex than that from cell lines due to the cellular heterogeneity and patient-specific difference of the samples. We aimed to identify *IGV_L_-IGJ_L_* sequences from the MMRF CoMMpass study (27, 59), a large, comprehensive and relatively uniform collection of data. Three CoMMpass samples were initially tested to evaluate the performance of the MiXCR analysis. Despite the potential complexity of the samples, MiXCR reliably identified a single *IGV_L_-IGJ_L_* clone from each of the three cases. These studies indicated that the major computational cost in the process depicted in **Figure 1B** was associated with the MiXCR assembly step. MiXCR is optimized for assembly of millions of clones from repertoire sequencing data (25). Assembly of many reads to a small number of clones, as was the goal here, is slow. In tests where all reads from single cases were used as the input to MiXCR processing times could exceed 24 h. We therefore asked whether reducing the number of input reads would allow assembly of contiguous *IGV_L_-IGJ_L_* clones in a more tractable time. Randomly selected samples of reads from three independent clinical cases from the CoMMpass data were used to test this approach. For each case, three independent, random samples at sizes of 100K, 250K, 500K, 1M, 2.5M, 5M, and 10M reads were used as the input to MiXCR. The processing time required for MiXCR to assemble clones increases non-linearly as a function of the number of input reads (**Figure 3A**), while processing additional reads did not substantially increase the length of the identified clonal sequence (**Figure 3B**) or the fraction of reads assigned to that sequence (**Figure 3C**). Therefore, a random sample of 5 million paired-end reads from the initial FASTQ file was found to be sufficient to reliably identify the major clonal *IGV_L_-IGJ_L_* sequences.

**Figure 4:**
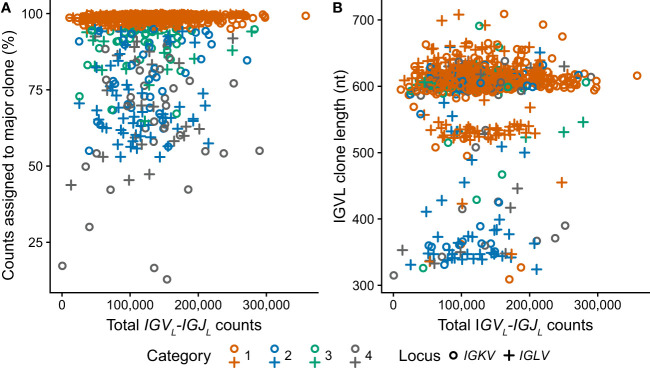
Clonal *IGV_L_-IGJ_L_* properties among 766 clinical samples are independent of the number of mapped reads. Fraction **(A)** and length **(B)** of the top *IGV_L_-IGJ_L_* clone are plotted against the total number of reads aligned to the *IGK* and *IGL* loci by MiXCR. Samples where the most frequent clone is derived from *IGKV* or *IGLV* are shown as circles and crosses, respectively. The category to which each clone was assigned is shown by the color of the symbol. For cases where the output from MiXCR comprised multiple non-contiguous segments for a single clonotype, only the longest segment was considered.

RNAseq data from 766 unique individuals at diagnosis of MM were downloaded from the restricted dbGaP resource, following approval. All data were successfully processed according to the scheme in **Figure 1B**. The median number of counts assigned to *IGV_L_-IGJ_L_* clones from 5M initial reads was 124,000 (2.5% of total reads, range 531-358,000). In most cases, a single clone accounted for more than half the assembled *IGKV-IGKJ* or *IGLV-IGLJ* read counts (**Figure 4A**), consistent with most CD138+ cells in the bone marrow sample being clonal MM plasma cells. At least 10,000 counts were assigned to the most frequent clone in all but one case, in which the largest *IGKV-IGKJ* clone was assigned only 90 counts (0.0018% of input reads). For this sample, the analysis was repeated using 50M initial reads to ensure that the lack of a clonal sequence was not due to insufficient sampling. The reanalysis identified the same *IGKV-IGKJ* clone with an identical CDR3 sequence, which was assigned 846 counts (0.0017% of input reads). Excluding this single clone, the most frequent clone was derived from *IGKV-IGKJ* in 486 cases, and from *IGLV-IGLJ* in 279 cases. In some cases, MiXCR identified two or more non-contiguous sequence fragments in the major clone. For these cases, only the longest fragment was considered for further analysis. The length of the major clonal sequence ranged between 309 and 709 nucleotides (nt), with a median length of 606 nt (**Figure 4B**). Due to the random sampling and biological variability, the total number of reads assigned to *IGK* and *IGL* loci varied between cases. Neither the fraction of counts assigned to the most frequent clone nor the length of that clone were related to the total number of *IGV_L_-IGJ_L_* counts identified from the initial sample of 5M reads (**Figure 4**), supporting our conclusion that 5M reads is sufficient to identify the clonal *IGV_L_-IGJ_L_* sequence.

### 3.3 Monoclonal sequences dominate the transcriptome of most clinical samples

**Figure 5:**
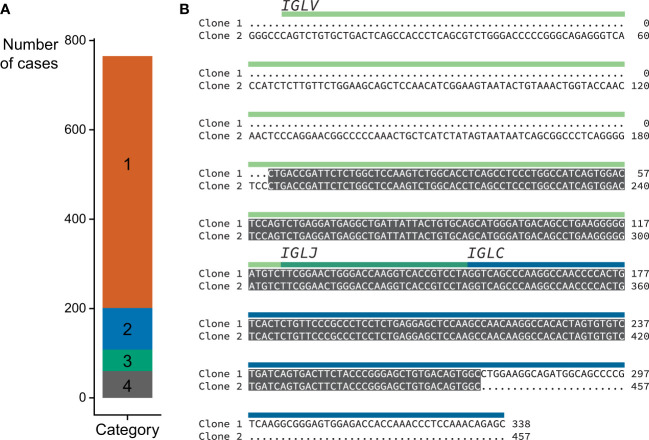
Locus and category assignment for 766 CoMMpass samples. **(A)** Sequences assigned to each category. Category colors are the same as in **Figure 4**. **(B)** Example Category 2 alignment comparing the top two clones identified by MiXCR within a single case (MMRF126178). The two clones are identical over a 254 nt region which includes CDR3, and were therefore collapsed to yield a single sequence. The regions corresponding to the precursor germline genes are shown with green and blue bars.

**Figure 6:**
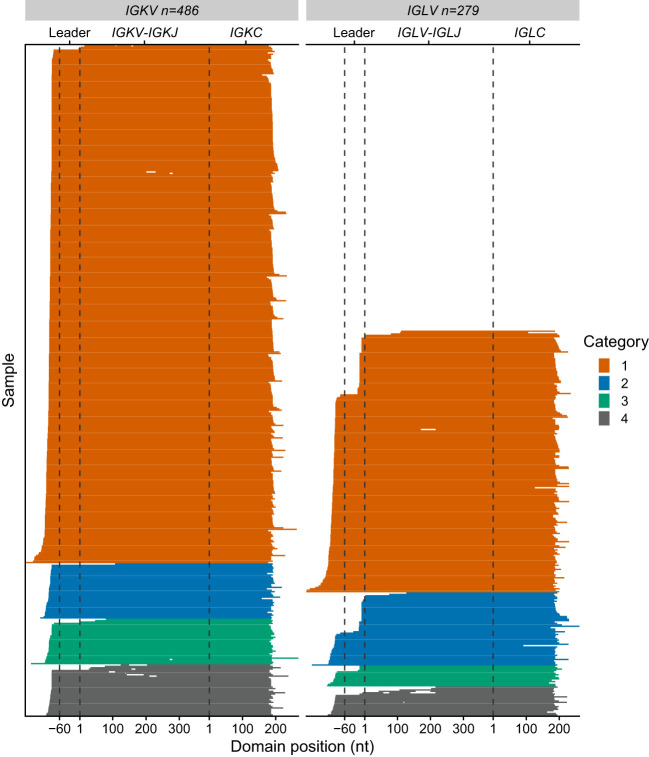
Sequence coverage for 765 *IGV_L_-IGJ_L_* clones. Each horizontal line represents a single clonotype sequence determined by MiXCR. Sequences are aligned according to the start of the *IGV_L_* and *IGC_L_* regions, which were identified by alignment to IMGT reference sequences. Gene regions are indicated with dashed lines. The first nucleotides of the *IGKV*/*IGLV* and *IGKC*/*IGLC* genes are used as reference points. Gaps represent regions of missing sequence; the differences in *IGV_L_-IGJ_L_* length between different germline genes are not shown. Colors represent the category to which each sequence is assigned, as for **Figures 4**, **5**.

To evaluate the performance of our pipeline on RNAseq data derived from primary patient samples, the 766 initial MMRF cases were divided into four categories based on the fraction of assembled counts that MiXCR assigned to the major clone (**Table 1** and **Figures 4**–**6**).

**Table 1:** Assignment of 766 samples to clonality categories.

	Assigned light chain clone (Complete *IGV_L_-IGJ_L_*)		
	*IGKV*	*IGLV*	Total
Category 1: ≥95% of counts assigned to clone	375 (370)	189 (183)	564 (553)
Category 2: Two clones with 100% identity over 200 nt that account for ≥95% of counts	40 (39)	53 (48)	93 (87)
Category 3: >50% of counts assigned to clone; ≤5% of counts assigned to next largest clone	33 (30)	15 (15)	48 (45)
Category 4: Samples which did not fulfil any of the above criteria	38 30	22 16	61* (46)
Total	486 (469)	279 (262)	766 (731)

The number of cases meeting the criteria for each category *One sample had too few LC counts for a clone to be assigned to a gene.

Category 1 (n = 564): In these cases, a clonal *IGV_L_-IGJ_L_* sequence was identified to which ≥95% of *IGL* or *IGK* counts were mapped.

Category 2 (n=93): In these samples a major clone could be identified, accounting for more than 50% but less than 95% of *IGV_L_-IGJ_L_* counts. Furthermore, a secondary clone with an identical sequence was identified by MiXCR such that the two clones together accounted for ≥95% of the total *IGV_L_-IGJ_L_* counts. Only pairs of clones without mismatches were considered for this category. The overlapping region was required to be ≥200 nt and include the CDR3 region. This behavior may be due to assignment of initial reads to distinct but similar germline genes. We collapsed these two clones to a single sequence. This procedure increased the median sequence length of Category 2 clones from 532 nt to 605 nt and the median fraction of counts associated with these clones from 72.7% to 98.9%. An example of one such alignment is shown in **Figure 5B**, where combining the two sequences was necessary to capture the complete *IGLV-IGLJ* sequence.

Category 3 (n=48), In these samples, the most frequent clone accounted for >50% of total *IGV_L_-IGJ_L_* counts, while the second most frequent clone accounted for <5% of counts. We did not attempt to collapse these clones into a single sequence.

Category 4 (n=61): Samples did not fit into the previous groups and were assigned to Category 4. For one sample, as described above, the largest clone was assigned only 90 counts, so the presence of a monoclonal *IGKV-IGKJ* or *IGLV-IGLJ* sequence was not determined. There were 13 samples in Category 4 where two clones with distinct CDR3 sequences each accounted for >10% of assigned counts. These samples may represent biclonal or oligoclonal disease.

**Figure 7:**
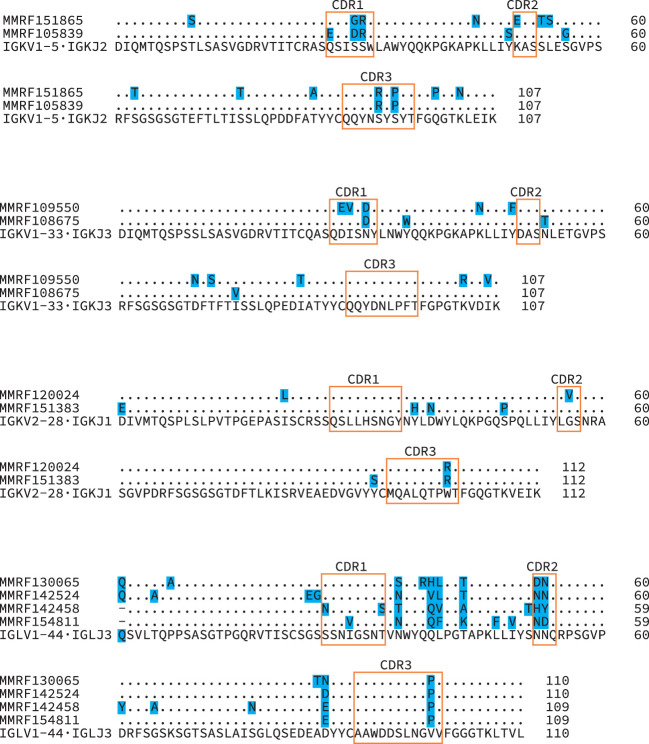
Diverse *IGV_L_-IGJ_L_* sequences despite identical CDR3 regions. Protein sequence alignments of four groups of clonal sequences where the CDR3 sequence is identical between clones. The *IGV_L_ and IGJ_L_* genes identified for all sequences within each group were identical, and the inferred protein sequences for these germline precursors are shown beneath the clonal sequences. Shaded residues highlight differences between sequences. Orange boxes indicate CDR regions, according to the IMGT classification. Numbers represent local position within the alignments.

Of the 705 sequences in Categories 1, 2 and 3 with a single major *IGV_L_-IGJ_L_* clone (448 *IGKV-IGKJ* and 257 *IGLV-IGLJ*), the sequences of 685 (97%) cover the complete *IGV_L_-IGJ_L_* region (**Figure 6** and **Table 1**). Each of the 705 clones encodes a unique LC protein sequence. However, 10 clones (0.14%) have a CDR3 nucleotide sequence that is shared by at least one other clone (**Figure 7**). There are four unique CDR3 nucleotide sequences among these 10 clones. Alignments of these clones reveal multiple residue differences outside CDR3, including in framework regions, supporting the hypothesis that they are distinct clones unique to each patient. In three out of four groups, analysis by IMGT V-Quest (57) identifies no mutations relative to the CDR3 region of the *IGKV* or *IGLV* gene. Accordingly, in these groups no amino acid residues differ from the germline sequences other than at the *IGV_L_-IGJ_L_* junction (**Figure 7**).

### 3.4 Clones identified by MiXCR are consistent with clinical data and previously determined sequences

**Figure 8:**
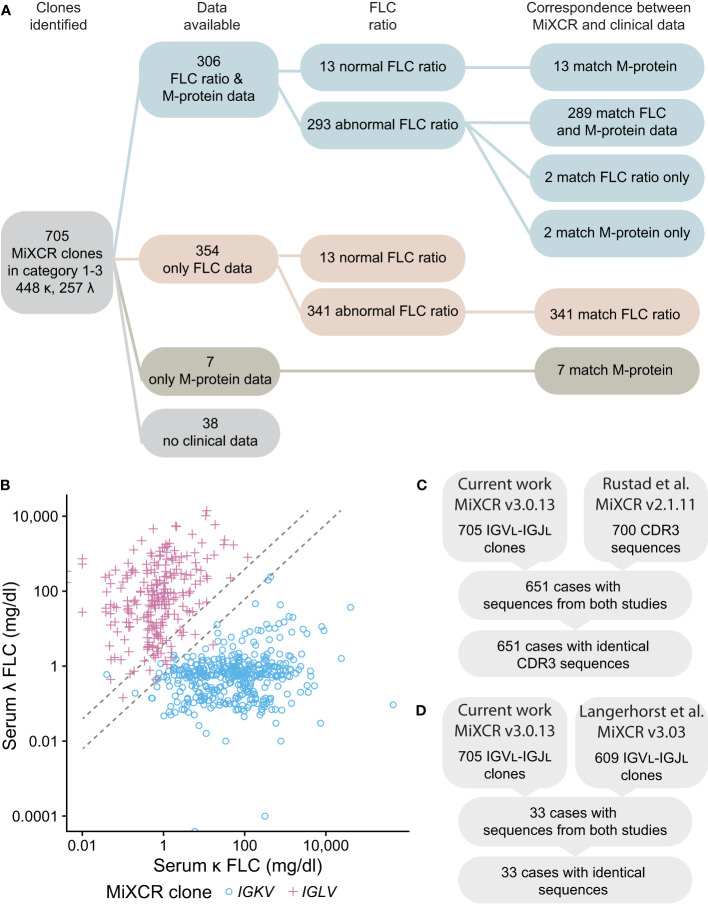
*IGV_L_-IGJ_L_* clones identified by MiXCR are consistent with clinical free LC ratios and previously determined sequences. **(A)** Workflow showing the number of samples available for comparison. Matching sequences are those where the IGK or IGL locus assigned by MiXCR is the same as that indicated by the clinical data. Only the identity of LC determined by immunofixation is shown for the M-protein data; of 313 samples with available M-protein results, 253 had complete immunoglobulin (data not shown). **(B)** Comparison of the κ to λ serum FLC ratio calculated from the CoMMpass clinical data with the identity of the clonal LC identified by MiXCR. *IGKV* and *IGLV* clones are shown as blue circles and pink crosses, respectively. Dashed lines demark the boundaries of normal κ to λ serum ratios. If the κ to λ serum ratio is >1.65 or <0.26, we would expect the most frequent MiXCR clone to be derived from *IGKV* or *IGLV*, respectively. **(C)** CDR3 sequences from Categories 1-3 determined in this work are identical to those previously determined by Rustad et al. (23). **(D)** *IGV_L_-IGJ_L_* sequences from Categories 1-3 determined in this work are identical to those previously determined by Langerhorst et al. (24). Only a representative example of sequences determined by Langerhorst et al. was available for comparison.

We next asked whether the clonal sequences determined from the RNAseq data are consistent with clinical data from the MMRF CoMMpass study. We compared the identity of the most frequent *IGV_L_-IGJ_L_* clone identified by MiXCR to serum FLC ratios measured at diagnosis. For the 634 samples where both an abnormal FLC ratio and MiXCR clone in Categories 1-3 were identified, the identity of this clone corresponded to the major FLC restriction type in 632 (99.7%) cases (**Figures 8A, B**). In two cases, MiXCR clone assignments were inconsistent with the FLC ratio. However, in both cases, the M-protein LC restriction identified in the clinical data was discordant with the FLC ratio and instead matched the MiXCR assignment. There were 26 cases where the FLC ratio was within the normal range. M-protein LC restriction data were available for 13 of these cases and in each instance the clinical LC restriction was consistent with the *IGV_L_-IGJ_L_* sequence identified by MiXCR. A further 7 cases had only M-protein data, all of which were consistent with the clone assigned by MiXCR.

Finally, the sequences identified in the current study were compared to those previously reported by studies of the CoMMpass data (23, 24). Six hundred and fifty-one of 700 *IGV_L_-IGJ_L_* CDR3 sequences from Rustad et al. (23) and 33 of 609 partial *IGV_L_-IGJ_L_* sequences from Langerhorst et al. (24) could be compared with the 705 sequences in Categories 1-3. All sequences compared were identical (**Figures 8C, D**). We therefore conclude that the *IGV_L_*-*IGJ_L_* sequences identified by MiXCR correspond to the PCD-associated monoclonal sequence.

### 3.5 Deposition of sequences in AL-Base

Clonal sequences from samples in Categories 1, 2 and 3 have been deposited to AL-Base (12) and are provided in the supplemental data. For 14 sequences, AL amyloidosis is reported in the clinical data, so these are assigned to the “AL-PCD” category and “AL/MM” subcategory. The *IGV_L_* germline gene assignment and clone category for these cases is shown in **Table 2**. The usage of germline genes by the *IGV_L_-IGJ_L_* sequences was similar to previously reported in AL amyloidosis (12, 13, 60), and the majority of clones from these cases were assigned to Category 1. There were no reported cases of amyloidosis among clones assigned to Category 4. Other 691 sequences are assigned to the “Other-PCD” category and “MM” subcategory.

**Table 2:** The *IGV_L_* germline gene usage and clone category in MM cases with AL amyloidosis.

Sample ID	IGV_L_ germline gene donor	Clone category
MMRF130770	IGKV1-33	3
MMRF199445	IGKV1-39	1
MMRF127029	IGKV1-39	1
MMRF115036	IGKV1-39	2
MMRF188808	IGKV3-15	1
MMRF194082	IGKV3-20	1
MMRF160272	IGKV4-1	1
MMRF110862	IGLV1-40	1
MMRF137305	IGLV1-47	1
MMRF162504	IGLV1-51	1
MMRF178661	IGLV2-14	2
MMRF198221	IGLV2-14	1
MMRF124805	IGLV3-1	3
MMRF127938	IGLV6-57	1

Complete *IGV_L_-IGJ_L_* sequences are available in the **Supplementary Information**.

## 4 Discussion

Here, we report a computational approach (**Figure 1B**) to identify clonal *IGV_L_-IGJ_L_* sequences from untargeted RNAseq data, based on the freely available MiXCR suite of tools (22, 25, 26). This approach is distinct from that taken by repertoire sequencing studies, which use targeted deep sequencing (10, 11), and studies that quantify clonal immune cells from RNAseq data based on clonal CDR3 sequences (22).

As an initial validation, we used published data derived from the U266 MM cell line (54, 55). The dominant U266 *IGLV2-8-IGLJ2* sequence determined by MiXCR from untargeted RNAseq data was identical to that previously cloned by standard methods over the region identified by both approaches (**Figure 2**). To optimize analysis of multiple samples, we tested how many input reads were required to confidently define a clonal sequence within a practical timeframe (**Figure 3**), identifying 5M reads as optimal for the 2x100 nt paired-end data studied here. For experiments where longer reads are recorded, fewer reads may be needed to define a clonal sequence. This analysis can be run on a typical personal computer for individual samples, or is suitable for multiplexed analysis of many samples on a cluster or cloud-based system. RNAseq experiments typically aim to acquire at least 30 million reads per sample for gene expression studies (see for example https://www.encodeproject.org/data-standards/encode4-bulk-rna/), so the number of reads available for analysis is unlikely to limit future studies. The high proportion of clonal sequences derived from these CD138-selected samples suggests that this approach may also be applicable to RNAseq data derived from total bone marrow samples.

We applied this approach to the MMRF CoMMpass dataset, comprising 766 cases with RNAseq data derived from CD138-selected bone marrow cells collected at diagnosis of MM (27). Despite the complexity of these clinical transcriptomes compared to that of a cell line, analysis of 5 million reads is sufficient to identify a clonal *IGV_L_-IGJ_L_* sequence in 705/766 cases. Any approach to identifying a single nucleotide sequence from a complex biological sample requires a balance between the desired precision and accuracy, and the effort needed to verify each sequence. Here, we attempted to maximize the sequence coverage of the clones but imposed strict parameters on which clones were accepted as representing the monoclonal sequence. To assess the performance of the analysis over multiple samples, we defined four categories that describe the frequency of the major clone. Categories 1, 2 and 3 define a major clonal *IGV_L_-IGJ_L_* sequence as accounting for >50% of the read counts assigned by MiXCR where the next most frequent clone accounts for <5% of counts (**Figure 4**). Of the 705 samples with a major clone, that clone accounts for >95% of counts in 657 samples, including 93 where MiXCR identified two apparently identical clones that we collapsed to a single sequence (i.e., Categories 1 and 2). All 705 *IGV_L_-IGJ_L_* sequences were unique to the clinical case. Ten clones had CDR3 nucleotide sequences that were shared with at least one other sequence, but had multiple sequence changes elsewhere in the *IGV_L_-IGJ_L_* region (**Figure 7**). Non-unique CDR3 sequences were also reported by Rustad et al. (23), who previously analyzed the MMRF CoMMpass data using MiXCR to identify CDR3 regions and showed that the *IGV_L_-IGJ_L_* CDR3 region could be used to track clones across samples. Previous studies have reported that specific *IGV_L_-IGJ_L_* clonotypes can be identified in multiple individuals (11, 61). These observations support the hypothesis that identifying complete *IGV_L_-IGJ_L_* sequences is necessary to understand the behavior of clonal FLCs.

There are 61 samples in Category 4, for which no major clone was identified. Because the purpose of this study is to identify dominant clones that can be used in other studies, we have not investigated these samples further. However, we speculate that these results could be due to one or more of the following factors. The 13 samples in Category 4 where the two largest clones have distinct CDR3 sequences may represent biclonal or oligoclonal disease, where two or more unique monoclonal proteins are identified in serum. The existence of two or more distinct M-protein or FLC clones in PCDs is not well understood, since immunological methods cannot distinguish between similar proteins. Identifying distinct clones from nucleotide sequences is an active area of investigation that is explored in more detail by Rustad et al. and Langerhorst et al. (23, 24). Minor clones with *IGV_L_-IGJ_L_* sequences related to the major clone may result from subclonal evolution of the PCD clone (62). Alternatively, the additional clones could represent either varying numbers of PCR errors during library preparation, or biological variability, such as the presence of non-clonal, healthy plasma cells or other B cells in the bone marrow sample.

Finally, we verified that the clone identified by MiXCR is consistent with the LC restriction type determined by serum FLC and/or M-protein testing in the patient from whom the original bone marrow sample was taken (**Figure 8**). The identity of our clones was confirmed by comparison to sequences reported in the studies of Rustad et al. and Langerhorst et al. (23, 24). For all evaluable cases, the sequences were identical to those previously published. All three studies therefore identified the same clonal sequences, irrespective of the number of input reads or the parameters used for MiXCR processing. Overall, each study demonstrates the utility of MiXCR to analyze data from PCD samples for different purposes: while the previous reports aimed to identify clones for disease monitoring, the goal of our study was to determine complete *IGV_L_-IGJ_L_* sequences to study physicochemical properties of LCs.

Identification of sequence features that influence pathologic aggregation of LCs could allow potentially aggregation-prone LCs to be detected before the onset of symptoms. AL amyloidosis and MM are invariably preceded by an asymptomatic phase known as monoclonal gammopathy of undetermined significance (63), during which a clonal FLC could potentially be investigated. Several studies have looked for LC sequence features that correlate with AL amyloidosis or other PCDs (18, 60, 64, 65), including the AL-Base resource, a curated database of LC sequences (12). Two machine learning algorithms, LICTOR and V_L_AmY-Pred, have been recently proposed to predict the amyloidogenic potential for a LC protein sequence (66, 67). However, efforts to develop prediction tools are hampered by the small number of available sequences associated with PCDs, relative to the vast potential sequence space. Established methods of cloning and sequence determination are slow (16, 17), limiting the number of monoclonal sequences that can be studied, so our method will allow additional sequences to be included in future studies. This approach is complementary to other recently developed methods that have used targeted amplification of *IGV_L_-IGJ_L_-IGC_L_* mRNA to create libraries for high throughput sequencing techniques (18, 19). The current work has increased the number of MM-associated sequences in AL-Base from 180 to 871 and the number of MM-associated sequences with observed amyloid formation from 29 to 43. We expect that future studies will benefit from the larger set of non-AL-associated monoclonal LC sequences reported here.

This study has several limitations. Only part of the *IGC_L_* region was identified by MiXCR, since MiXCR is designed to focus on *IGV_L_-IGJ_L_* gene regions. We anticipate that the full *IGC_L_* sequence could be retrieved from RNAseq data by using another aligner program and combining the resulting sequences. However, it is challenging to ensure that a standard aligner, which is not optimized for immunoglobulins, is able to correctly identify the clone associated with any mutations detected in the *IGC_L_* region, so integrating this step into our pipeline was beyond the scope of the current work. Identification of the complete *IGC_L_* region may also be possible using sequencing platforms that acquire longer reads. Identifying full-length *IGC_L_* sequences would address the roles of the LC constant domain in amyloidosis and other disorders (68–72).

A more significant limitation is that the classification of clinical cases as having amyloidosis likely underestimates the frequency of amyloid fibrils, which may be present in up to one third of MM cases without reaching the levels necessary to cause organ dysfunction (73–75). Only 14/705 clones (2.0%) were annotated as having amyloidosis in the CoMMpass study (**Table 2**). Most LC proteins appear to be able to form amyloid under some conditions, so both the concentration of FLC in circulation and biophysical properties appear to influence amyloid deposition (1, 15). FLC levels vary widely between patients in the MMRF cohort (**Figure 8B**), while the majority of these clones primarily secrete a complete immunoglobulin M-protein, as described in previous studies (23, 24). Though complete immunoglobulins may be less prone to aggregation, it is not possible to exclude that MM-associated sequences could have amyloidogenic potential and therefore aggregate in patients if produced at a higher level in FLC form. Therefore, these sequences should be considered as “less prone to aggregate” than sequences known to be associated with AL amyloidosis, rather than “non-aggregating”.

Two factors that correlate with risk of systemic AL amyloidosis are a lack of heavy chain partner and the particular germline precursor gene usage by amyloidogenic *IGV_L_-IGJ_L_* sequence. AL amyloidosis appears to occur in 5-10% of cases of LC-only MM (LCMM) that is characterized by inability of clonal cells to produce heavy chain, resulting in the exclusive production of FLC (76, 77). Half of patients diagnosed with AL amyloidosis in published studies have no detectable complete immunoglobulin M-protein, compared to around one in six patients with MM (77, 78). Of 313 MMRF samples in categories 1-3 with available M-protein data, 60 (19.2%) had no complete immunoglobulin (not shown). This is consistent with previous reports (77) and may indicate LCMM cases that would be at increased risk of amyloid formation. Furthermore, although most MM patients develop kidney injury (7), which is more common in LCMM (77), the presence of renal amyloid fibrils or amorphous deposits is not always investigated *via* renal biopsies.

In addition, clonal sequences derived from the *IGLV6-57* precursor gene are strongly associated with AL amyloidosis (12, 60, 79). Eight of 705 (1.1%) clones were derived from *IGLV6-57* and one of these sequences (MMRF127938) was reported to have amyloidosis. A review of the available CoMMpass clinical data for the remaining 7 cases revealed one individual with impaired renal function, which may be due to either AL amyloidosis or MM. Two cases had evidence of asymptomatic non-progressive Grade 1 peripheral neuropathy that could likely be attributed to MM rather than AL amyloidosis. Further information on cardiac, hepatic or soft tissue features that are commonly seen in AL amyloidosis was not present in the data. Congo red staining of biopsied tissue would be required to diagnose AL amyloidosis, which would necessitate modification of treatment regimens (80). Three *IGLV6-57* derived MM sequences have been previously reported in AL-Base, of which one was associated with AL amyloidosis (17). Relevant clinical follow-up information is not available for two further sequences (81, 82).

In conclusion, this work provides a straightforward approach to determining *IGV_L_-IGJ_L_* sequences from new or existing RNAseq data. We anticipate that RNAseq experiments will increasingly become part of routine clinical practice to investigate both symptomatic and asymptomatic PCDs, so our MiXCR-based pipeline will allow *IGV_L_-IGJ_L_* sequences identified from these data to be investigated for a range of clinical applications. This work increases the number of monoclonal *IGV_L_-IGJ_L_* sequences known to be associated with MM and available to the wider research community *via* AL-Base by over four-fold. These sequences and those determined in future studies will facilitate investigations into the mechanisms of FLC aggregation in PCDs.

## Data availability statement

The data analyzed in this study is subject to the following licenses/restrictions: RNAseq data are deposited in dbGaP and require an application for access. Associated clinical data are available by application to the MMRF. Requests to access these datasets should be directed to https://www.ncbi.nlm.nih.gov/projects/gap/cgi-bin/study.cgi?study_id=phs000748.v7.p4.

## Author contributions

GJM, TP and VS manage the AL-Base resource and the clinical data therein, and obtained funding for this project. GJM conceived the project. AN carried out the research work with assistance from other authors. YS provided technical assistance with computational resources. AN, TP and GJM wrote the manuscript. All authors contributed to the article and approved the submitted version.
